# Supplementary material for: Intervention Activities Associated with the Implementation of a Comprehensive School Tobacco Policy at Danish Vocational Schools: A Repeated Cross-Sectional Study
Source: Int J Environ Res Public Health. 2022 Sep 30;19(19):12489. doi: 10.3390/ijerph191912489 (PMC9565121; doi:10.3390/ijerph191912489)
Supplement: Supplementary file 1 [file ijerph-19-12489-s001.zip › Table S1.pdf]

1. How this paper adheres to the STROBE checklist for reporting cross-sectional studies

Supplementary Table S1: STROBE checklists for reporting cross-sectional studies, including how this study adheres to the checklist.

**The STROBE checklist for cross-sectional studies**				**How and where the paper adheres to the checklist**
Heading	Content	Item No	Checklist	
Title and abstract	NA	1	(a) Indicate the study's design with a commonly used term in the title or the abstract	The title and abstract indicate that we applied a repeated cross-sectional study design.
			(b) Provide in the abstract an informative and balanced summary of what was done and what was found	In the abstract, we summarize the key elements of the study as well as results.
Introduction	Background /rationale	2	Explain the scientific background and rationale for the investigation being reported	In the Introduction, we describe and discuss two interrelated research gaps and how the study aims to address them.
	Objectives	3	State specific objectives, including any pre-specified hypotheses	By the end of the Introduction, we describe the purpose of the study and the specific research question.
Methods	Context	*Not part of the checklist*		We added two sections about the context of the study, as we found them necessary to understand the study design: "Setting" describe the Danish vocational school system and "the Smoke-Free Vocational Schools intervention" describe the intervention.
	Study design	4	Present key elements of study design early in the paper	The key elements of the study design are presented under "Study design" i.e., the repeated cross-sectional study design, main variables of interest, and respondent groups.
	Setting	5	Describe the setting, locations, and relevant dates, including periods of recruitment, exposure, follow-up, and data collection	The sections "Study population" and "Data collection" covers the data collection procedures/recruitment, response rates, and timing as well as the eligibility (and exclusion criteria) for inclusion. Also, we reported the number of participants at each stage under "Study population" (not under Results).
	Participants	6	(*a*) Give the eligibility criteria, and the sources and methods of selection of participants	
	Variables	7	Clearly define all outcomes, exposures, predictors, potential confounders, and effect modifiers. Give diagnostic criteria, if applicable	All variables are presented, including outcomes "Implementation fidelity measures – independent variables", exposures "intervention activities – independent variables" and confounders "context – confounding variables. The description of possible effect-modifiers was added to the "context – confounding variables" section. All variables are described in detail and available for reproduction.
	Data sources/ measurement	8	For each variable of interest, give sources of data and details of methods of assessment (measurement). Describe comparability of	

			assessment methods if there is more than one group	
	Bias	9	Describe any efforts to address potential sources of bias	We do not explicitly address bias in the Methods section. Possible biases are discussed in the “Strengths and Limitations” section in the Discussion.
	Study size	10	Explain how the study size was arrived at	The final study size is explained under “Study population”.
	Quantitative variables	11	Explain how quantitative variables were handled in the analyses. If applicable, describe which groupings were chosen and why	The quantitative variables are explained under “Measures”.
	Statistical methods	12	(*a*) Describe all statistical methods, including those used to control for confounding	In the “Statistical analyses” section we describe all statistical methods, including those used to control for confounding:<br>- We describe the methods applied in the main statistical analyses (generalized linear mixed models) as well as the methods used to calculate the intra-class correlation coefficient.<br>- We describe the adjustment for confounders.<br>- We explain the methods applied in the sensitivity analysis (interaction analysis).<br>- We explain how missing data was handled.
			(*b*) Describe any methods used to examine subgroups and interactions	
			(*c*) Explain how missing data were addressed	
			(*d*) If applicable, describe analytical methods taking account of sampling strategy	
			(*e*) Describe any sensitivity analyses	
Results	Participants	13	(a) Report numbers of individuals at each stage of study—e.g., numbers potentially eligible, examined for eligibility, confirmed eligible, included in the study, completing follow-up, and analysed	The number of individuals is reported as part of the “Study population” section under Methods.
			(b) Give reasons for non-participation at each stage	Not applicable
			(c) Consider use of a flow diagram	Not applicable
	Descriptive data	14	(a) Give characteristics of study participants (e.g., demographic, clinical, social) and information on exposures and potential confounders	Table 4 (as well as Appendix 4) provides characteristics of the study population.
			(b) Indicate number of participants with missing data for each variable of interest	Appendix 2 provides information on missing data all analyses.
	Outcome data	15	Report numbers of outcome events or summary measures	Table 5 shows the describe results concerning the independent variables,

				whereas table 6 shows the describe results regarding the outcomes.
	Main results	16	(*a*) Give unadjusted estimates and, if applicable, confounder-adjusted estimates and their precision (eg, 95% confidence interval). Make clear which confounders were adjusted for and why they were included	The adjusted results, including 95% confidence interval, are reported in table 7a and table 7b including. The unadjusted results are available in Appendix 7.
			(*b*) Report category boundaries when continuous variables were categorized	Not applicable
			(*c*) If relevant, consider translating estimates of relative risk into absolute risk for a meaningful time period	Not applicable
	Other analysis	17	Report other analyses done—e.g., analyses of subgroups and interactions, and sensitivity analyses	Results from the sensitivity analysis are reported and the full analysis is available in Appendix 8.
Discussion	Key results	18	Summarize key results with reference to study objectives	The key results are summarized under "Key findings".
	Limitations	19	Discuss limitations of the study, taking into account sources of potential bias or imprecision. Discuss both direction and magnitude of any potential bias	The strengths and limitations, including potential biases are discussed under "Strengths and Limitations". Furthermore, we thoroughly discuss strengths and limitations of the operationalization of implementation outcomes under "Operationalization of policy implementation outcomes".
	Interpretation	20	Give a cautious overall interpretation of results considering objectives, limitations, multiplicity of analyses, results from similar studies, and other relevant evidence	In the "Interpretations of results" section, we discuss and position our results in relation to the extensive literature.
	Generalizability	21	Discuss the generalizability (external validity) of the study results	In the "Implications for research and practice" section, we discuss the generalizability of the study findings.
Conclusion	*Not part of the checklist*			We briefly summarize the main findings in the Conclusion.
Other information	Funding	22	Give the source of funding and the role of the funders for the present study and, if applicable, for the original study on which the present article is based	
