# Supplementary material for: Intervention Activities Associated with the Implementation of a Comprehensive School Tobacco Policy at Danish Vocational Schools: A Repeated Cross-Sectional Study
Source: Int J Environ Res Public Health. 2022 Sep 30;19(19):12489. doi: 10.3390/ijerph191912489 (PMC9565121; doi:10.3390/ijerph191912489)
Supplement: Supplementary file 1 [file ijerph-19-12489-s001.zip › Table S2.pdf]

2. Number of individuals (N) in each adjusted analysis

Table S2: Number of individuals (N) in each analysis at student level

	N in each adjusted* analysis**				
	**Adherence**	**Dose**	**Quality of delivery**	**Participant responsiveness**	**Total Implementation**
Student level - Time 1 (T1)					
New school-break facilities	1189	1189	1086	1189	1086
Smoke-free signage	1189	1189	1086	1189	1086
Help to deal with not smoking during school hours and smoking cessation assistance	365	365	321	365	1086
Student level - Time 2 (T1)					
New school-break facilities	1406	1401	1406	1406	1406
Smoke-free signage	1406	1401	1406	1406	1406
Help to deal with not smoking during school hours and smoking cessation assistance	381	379	381	381	381

* The analyses were adjusted for age, sex, smoking status, main subject area, and educational level. ** The variations in N are either due to missing responses to 'intervention activity variables or confounders.

Table S2: Number of individuals (N) in each analysis at staff/manager level

	N in each adjusted* analysis**				
	**Adherence**	**Dose**	**Quality of delivery**	**Participant responsiveness**	**Total implementation**
Staff/manager level - Time 1 (T1)					
Joint workshop before policy implementation	184	184	184	184	184
Internalization of fixed enforcement procedures	419	419	419	419	419
Experienced support from NGOs and local municipality	411	411	411	411	411
Staff/manager level - Time 2 (T1)					
Joint workshop before policy implementation	134	134	134	134	134
Internalization of fixed enforcement procedures	418	418	418	418	418
Experienced support from NGOs and local municipality	402	402	402	402	402

* The analyses were adjusted for age, sex, smoking status, and if staff/managers had a special function in relation to health promotion. ** The variations in N are either due to missing responses to 'intervention activity variables or confounders.
