# Supplementary material for: Intervention Activities Associated with the Implementation of a Comprehensive School Tobacco Policy at Danish Vocational Schools: A Repeated Cross-Sectional Study
Source: Int J Environ Res Public Health. 2022 Sep 30;19(19):12489. doi: 10.3390/ijerph191912489 (PMC9565121; doi:10.3390/ijerph191912489)
Supplement: Supplementary file 1 [file ijerph-19-12489-s001.zip › Table S3.pdf]

3. Independent variables, including contextual factors (confounders)

Table S3: Context variables (confounders)

Elements of context	Items	Response categories
Student level		
Educational track	What is your main subject area?	Care, health, and pedagogy/ Administration, commerce, and business service/ Technology, construction, and transportation/ Food, agriculture, and hospitality
Educational level	Are you enrolled at the normal vocational education program or the higher vocational education program?	Vocational school normal/ Vocational school higher
Sex	(Based on social security number)	Male/female
Age	(Based on social security number)	Age
Smoking status	Do you smoke cigarettes?	Yes, every day/ Yes, weekly/ Yes, but less than once a week/ No, I quitted smoking cigarettes/ No, but I have tried smoking cigarettes/ No, I have never smoked cigarettes - not even a puff
Staff/manager level		
Special function in relation to health promotion	Do you have a special function in relation to health promotion or well-being at this school?	No / I am smoking cessation counsellor / I am well-being consultant / I am part of the schools' health team / I am responsible for school health / I am a mentor / I am a contact teacher / I am school psychologist / I am the staff representative / I am the occupational health representative / Other function [open question]
Sex	Are you male or female?	Male/female
Age	How old are you?	Drop-down with ages: 18-75
Smoking status	Do you smoke cigarettes?	Yes, every day/ Yes, weekly/ Yes, but less than once a week/ No, I quitted smoking cigarettes/ No, but I have tried smoking cigarettes/ No, I have never smoked cigarettes - not even a puff
