# Supplementary material for: Intervention Activities Associated with the Implementation of a Comprehensive School Tobacco Policy at Danish Vocational Schools: A Repeated Cross-Sectional Study
Source: Int J Environ Res Public Health. 2022 Sep 30;19(19):12489. doi: 10.3390/ijerph191912489 (PMC9565121; doi:10.3390/ijerph191912489)
Supplement: Supplementary file 1 [file ijerph-19-12489-s001.zip › Table S4.pdf]

4. Study population characteristics stratified by intervention schools and by smoking status

Table S4: Characteristics of the student study population at T1 and T2, stratified by the intervention schools

	**Total population**	**School 1**	**School 2**	**School 3**	**School 4**	**School 5**	**School 6**	**School 7**
Student level characteristics - Time 1 (T1)								
N individuals	1222	130	259	117	103		75	128
(%)	(100)	(10.6)	(21.2)	(9.6)	(8.4)	410 (33.8)	(6.1)	(10.5)
Age, mean	23.5	23.8	29.8	18.7	20.4	22.1	26.2	20.5
(SD)	(9.3)	(8.5)	(11.6)	(0.9)	(8.6)	(7.5)	(10.5)	(6.5)
Male gender %	59.0	80.8	17.1	53.8	58.3	84.1	14.7	72.7
Main subject area %								
Care, health, and pedagogy	29.2	0	100	0	14.0	0	100	0
Administration, commerce, and business service	17.9	24.6	0	100	63.0	1.2	0	4.0
Food, agriculture, and hospitality	5.8	1.7	0	0	4.0	6.4	0	28.0
Technology, construction, and transportation	47.1	73.7	0	0	19.0	92.4	0	68.0
Educational level %								
Vocational school-normal	74.0	83.9	83.3	22.6	31.0	88.0	84.0	76.0
Smoking prevalence %	30.5	40.0	29.7	25.6	30.1	28.5	24.0	37.5
Student level characteristics - Time 2 (T2)								
N individuals	1452		234	300	120		146	128
(%)	(100)	152 (10.5)	(16.6)	(20.7)	(8.3)	372 (25.6)	(10.1)	(8.8)
Age, mean	22.5	24.0	26.0	19.3	20.0	22.0	28.4	21.1
(SD)	(8.5)	(8.5)	(10.9)	(3.4)	(9.2)	(5.4)	(12.1)	(6.7)
Male gender %	58.5	83.6	13.7	52.0	52.4	84.1	22.7	89.0
Main subject area %								
Care, health, and pedagogy	28.2	0	100	0	17.4	0	100	4.3
Administration, commerce, and business service	27.7	7.9	0	100	72.2	1.0	0	1.7
Food, agriculture, and hospitality	7.0	18.5	0	0	2.6	9.1	0	27.4
Technology, construction, and transportation	37.0	71.5	0	0	7.8	89.9	0	66.7
Educational level %								
Vocational school-normal	66.0	88.1	78.2	18.4	24.1	87.9	84.8	84.6
Smoking prevalence %	27.3	32.9	26.5	28.3	23.3	28.2	20.5	28.9

Supplementary Table S4: Characteristics of the staff/manager study population at T1 and T2, stratified by the intervention schools

	**Total population**	**School 1**	**School 2**	**School 3**	**School 4**	**School 5**	**School 6**	**School 7**
Staff/manager level characteristics - Time 1 (T1)								
N individuals	419	72	102	46	47	77	24	51
(%)	(100)	(17.2)	(24.3)	(11.0)	(11.2)	(18.4)	(5.7)	(12.2)
Age, mean	48.6	50.8	49.9	47.7	43.9	51.1	50.3	44.1
(± SD)	(± 9.8)	(± 9.3)	(± 9.6)	(± 7.9)	(± 9.9)	(± 9.3)	(± 9.6)	(± 10.6)
Male gender, %	42.7	45.8	23.5	32.6	42.6	66.2	29.2	56.9
School position, %								
Manager	11.0	13.9	8.8	8.7	14.9	6.5	12.5	15.7

Teacher	62.8	54.2	58.8	52.2	63.8	79.2	62.5	66.7
Counsellor	6.7	1.4	7.8	13.0	10.6	5.2	4.2	5.9
Administrative	11.7	16.7	17.6	15.2	8.5	6.5	8.3	2.0
Other positions	7.9	13.9	6.9	10.9	2.1	2.6	12.5	9.8
Special function in relation to health promotion*, %	47.5	33.3	52.9	34.8	51.1	57.1	58.3	45.1
Smoking prevalence, %	12.9	15.3	10.8	10.9	14.9	5.2	29.2	17.6
Staff/manager level characteristics - Time 2 (T2)								
N individuals (%)	452 (100)	85 (18.8)	102 (22.6)	45 (10.0)	49 (10.8)	91 (20.1)	33 (7.3)	47 (10.4)
Age, mean (± SD)	46.9 (± 10.2)	51.1 (± 10.1)	47.1 (± 10.0)	48.6 (± 10.3)	42.9 (± 9.4)	49.2 (± 8.9)	43.0 (± 9.4)	40.4 (± 10.0)
Male gender, %	42.7	39.0	23.9	52.2	50.0	65.1	9.1	81.8
School position, %								
Manager	5.7	9.8	5.6	8.7	0.0	7.0	4.5	0.0
Teacher	65.9	63.4	54.9	52.2	83.3	72.1	72.7	81.8
Counsellor	6.9	2.4	9.9	13.0	0.0	4.7	13.6	4.5
Administrative	11.0	14.6	15.5	8.7	8.3	9.3	9.1	0.0
Other positions	10.6	13.9	14.1	17.4	8.3	7.0	9.1	4.5
Special function in relation to health promotion*, %	47.0	50.0	42.7	45.5	31.9	50.0	68.2	52.2
Smoking prevalence, %	12.4	10.6	6.9	15.6	18.4	13.2	12.1	17.0

** At both time points, the special functions mostly included 'contact teachers' i.e., a person who the students can contact in relation to both educational goals and personal issues (approx. 30 %).*

Table S4: Student study population at T1 and T2 stratified by smoking status

	**Smokers**	**Nonsmokers**
Student level characteristics - Time 1 (T1)		
N individuals (%)	373 (100)	848 (100)
Age, mean (SD)	22.9 (7.5)	23.8 (9.9)
Male gender, %	60.5	58.4
Educational track, %		
Care, health, and pedagogy	28.1	29.7
Administration, commerce, and business service	16.9	18.3
Food, agriculture, and hospitality	6.8	5.3
Technology, construction, and transportation	48.1	46.7
Educational level, %		
Vocational school-normal	80.1	71.3
Student level characteristics - Time 2 (T2)		
N individuals (%)	397 (100)	1055 (100)
Age, mean (± SD)	22.6 (7.6)	22.4 (8.8)
Male gender, %	57.7	58.8
Educational track, %		
Care, health, and pedagogy	25.0	29.5
Administration, commerce, and business service	27.9	27.7
Food, agriculture, and hospitality	6.8	7.0
Technology, construction, and transportation	40.3	35.8
Educational level, %		
Vocational school-normal	68.5	65.0
