# Supplementary material for: Intervention Activities Associated with the Implementation of a Comprehensive School Tobacco Policy at Danish Vocational Schools: A Repeated Cross-Sectional Study
Source: Int J Environ Res Public Health. 2022 Sep 30;19(19):12489. doi: 10.3390/ijerph191912489 (PMC9565121; doi:10.3390/ijerph191912489)
Supplement: Supplementary file 1 [file ijerph-19-12489-s001.zip › Table S5.pdf]

5. Intervention activity results stratified by intervention schools

Table S5: Student level intervention activity results at T1 and T2, stratified by the intervention schools

**Intervention activities***	**Total** mean (SD)	**School 1** mean (SD)	**School 2** mean (SD)	**School 3** mean (SD)	**School 4** mean (SD)	**School 5** mean (SD)	**School 6** mean (SD)	**School 7** mean (SD)
Student level - time 1 (T1)								
New school-break facilities (n=1119)	2.82 (1.1)	3.00 (1.1)	3.21 (1.1)	2.50 (1.0)	2.58 (1.1)	2.64 (0.9)	3.51 (1.0)	2.63 (1.1)
Smoke-free signage (n=1116)	2.58 (1.3)	2.73 (1.3)	3.15 (1.3)	2.53 (1.2)	1.94 (1.2)	2.47 (1.3)	3.11 (1.3)	1.91 (1.1)
Help to deal with not smoking during school hours and smoking cessation assistance (n=373)	2.01 (1.2)	2.17 (1.2)	2.48 (1.4)	1.95 (1.2)	2.33 (1.3)	1.72 (1.0)	1.63 (1.2)	1.78 (1.0)
Student level - time 2 (T2)								
New school-break facilities (n=1443)	2.91 (1.2)	2.85 (1.1)	3.23 (1.0)	2.63 (1.0)	2.90 (0.9)	2.72 (1.1)	3.49 (1.1)	2.94 (1.1)
Smoke-free signage (n=1440)	2.52 (1.3)	2.73 (1.2)	2.95 (1.4)	2.34 (1.2)	2.24 (1.4)	2.56 (1.3)	2.83 (1.4)	1.66 (1.4)
Help to deal with not smoking during school hours and smoking cessation assistance (n=397)	1.73 (1.0)	1.73 (1.0)	1.83 (0.9)	2.17 (1.3)	1.78 (0.9)	1.40 (0.7)	1.81 (1.0)	1.36 (0.6)

**All intervention activity variables are assessed on Likert scales from 1-5.*

Table S5: Staff/manager level intervention activity results at T1 and T2, stratified by the intervention schools

**Intervention activities***	**Total** mean (SD)	**School 1** mean (SD)	**School 2** mean (SD)	**School 3** mean (SD)	**School 4** mean (SD)	**School 5** mean (SD)	**School 6** mean (SD)	**School 7** mean (SD)
Staff/manager level - Time 1 (T1)								
Joint workshop before policy implementation (n=184)	3.35 (0.9)	3.20 (0.9)	3.09 (0.8)	3.53 (0.8)	3.07 (0.9)	N/A**	3.64 (0.8)	3.91 (0.8)
Internalization of fixed enforcement procedures (n=419)	3.07 (1.1)	2.76 (1.1)	3.25 (0.9)	3.39 (1.9)	3.23 (1.1)	2.45 (1.1)	2.88 (0.7)	3.69 (1.0)
Experienced support from NGOs and local municipality (n=411)	3.46 (0.9)	3.40 (0.9)	3.52 (0.8)	3.68 (0.8)	3.62 (0.8)	3.16 (0.9)	3.48 (0.8)	3.53 (1.0)
Staff/manager level - Time 2 (T2)								
Joint workshop before policy implementation (n=134)	3.35 (0.9)	3.13 (0.8)	3.20 (0.9)	3.57 (0.9)	3.04 (0.9)	N/A**	N/A***	3.93 (0.7)
Internalization of fixed enforcement procedures (n=452)	2.98 (1.0)	2.56 (0.9)	3.07 (1.0)	3.20 (1.0)	3.29 (0.9)	2.73 (1.1)	2.67 (0.9)	3.72 (0.9)
Experienced support from NGOs and local municipality (n=436)	3.36 (0.8)	3.42 (0.8)	3.35 (0.8)	3.55 (0.9)	3.25 (0.9)	3.26 (0.8)	3.28 (0.7)	3.44 (0.7)

**All intervention activity variables are assessed on Likert scales from 1-5. **The* joint workshop *did not take place at school 5.*
****No respondents at T2 at school 6 had participated in the joint workshop.*
