# Supplementary material for: Intervention Activities Associated with the Implementation of a Comprehensive School Tobacco Policy at Danish Vocational Schools: A Repeated Cross-Sectional Study
Source: Int J Environ Res Public Health. 2022 Sep 30;19(19):12489. doi: 10.3390/ijerph191912489 (PMC9565121; doi:10.3390/ijerph191912489)
Supplement: Supplementary file 1 [file ijerph-19-12489-s001.zip › Table S6.pdf]

6. Implementation fidelity results stratified by intervention schools

Table S6: Student level implementation fidelity results, stratified by intervention schools, at time 1 and 2 – Proportion of 'implemented'.

**Fidelity measures**	**Total** %	**School 1** %	**School 2** %	**School 3** %	**School 4** %	**School 5** %	**School 6** %	**School 7** %
Student level - Time 1 (T1)								
Adherence	87.3	87.7	90.0	88.9	91.3	81.1	97.3	90.6
Dose	28.2	34.6	25.9	32.5	21.4	25.6	36.0	31.3
Quality of delivery	91.1	37.0**	93.1	96.6	92.2	89.0	97.3	95.3
Participant responsiveness	25.3	13.1	25.9	31.6	18.4	22.4	33.3	40.6
Total implementation fidelity, mean* (SD)	2.24 (0.8)	1.43 (0.7)	2.34 (0.7)	2.49 (0.8)	2.23 (0.7)	2.18 (0.8)	2.64 (0.8)	2.57 (0.7)
Student level - Time 2 (T2)								
Adherence	86.8	86.8	89.3	91.3	92.5	80.9	87.0	82.8
Dose	32.7	28.9	34.2	30.7	28.3	27.2	45.2	45.3
Quality of delivery	92.6	94.9	94.9	96.7	95.8	87.6	97.3	94.5
Participant responsiveness	28.4	9.9	33.8	31.7	35.8	21.8	32.9	40.6
Total implementation fidelity, mean* (SD)	2.36 (0.8)	2.05 (0.7)	2.49 (0.8)	2.47 (0.7)	2.50 (0.7)	2.12 (0.9)	2.58 (0.9)	2.54 (1.0)

** Total implementation fidelity is the sum across fidelity measures (range: 0-4). ** N=27 on this specific item at T1 due to a survey technical malfunction (N was supposed to be 130).*

Table S6: Staff/manager level implementation fidelity results, stratified by the intervention schools, at time 1 and 2 – Proportion of 'implemented'.

**Fidelity measures**	**Total** %	**School 1** %	**School 2** %	**School 3** %	**School 4** %	**School 5** %	**School 6** %	**School 7** %
Staff/manager level - Time 1 (T1)								
Adherence	93.6	83.3	99.0	97.8	95.7	87.0	100	98.0
Dose	65.4	44.4	71.6	78.3	76.6	53.2	75.0	74.5
Quality of delivery	70.6	80.6	73.5	73.9	63.8	70.1	52.9	75.0
Participant responsiveness	51.1	43.1	35.3	69.6	63.8	39.0	54.2	82.4
Total implementation fidelity, mean* (SD)	2.80 (0.8)	2.51 (0.9)	2.79 (0.7)	3.19 (0.8)	3.00 (0.8)	2.49 (0.7)	3.04 (0.7)	3.07 (0.6)
Staff/manager level - Time 2 (T2)								
Adherence	93.8	88.2	97.1	88.9	98.0	92.3	93.8	100
Dose	68.6	44.7	67.6	84.4	83.7	70.3	72.3	78.8
Quality of delivery	62.8	75.3	62.7	53.3	59.2	68.1	54.5	48.9
Participant responsiveness	67.7	51.8	67.6	77.8	81.6	59.3	75.8	83.0
Total implementation fidelity, mean* (SD)	2.92 (0.7)	2.60 (0.7)	2.95 (0.7)	3.04 (0.8)	3.22 (0.6)	2.90 (0.7)	3.03 (0.8)	3.04 (0.6)

** Total implementation fidelity is the sum across fidelity measures (range: 0-4).*
