# Supplementary material for: Intervention Activities Associated with the Implementation of a Comprehensive School Tobacco Policy at Danish Vocational Schools: A Repeated Cross-Sectional Study
Source: Int J Environ Res Public Health. 2022 Sep 30;19(19):12489. doi: 10.3390/ijerph191912489 (PMC9565121; doi:10.3390/ijerph191912489)
Supplement: Supplementary file 1 [file ijerph-19-12489-s001.zip › Table S7.pdf]

7. Crude results

Table S7: Unadjusted associations between intervention activities and implementation fidelity of the smoke-free school hours policy, across T1 and T2, at student level.

	Odds Ratio (OR) with 95% confidence interval (95% CI)<br>P-value				Linear effect with 95 CI<br>P-value
	**Adherence**	**Dose**	**Quality of delivery**	**Participant responsiveness**	**Total implementation fidelity**
Student level - time 1					
New school-break facilities (n=1116-1219) *	1.11 [0.96-1.29]<br>0.142	**1.14 [1.02-1.27]<br>0.013**	1.19 [0.98-1.43]<br>0.067	1.11 [0.99-1.25]<br>0.063	**0.07 [0.03-0.11]<br>0.000**
Smoke-free signage (n=1116-1219) *	**1.39 [1.21-1.60]<br>0.000**	0.95 [0.86-1.04]<br>0.318	1.17 [0.99-1.39]<br>0.052	0.93 [0.84-1.03]<br>0.191	0.02 [-0.009-0.06]<br>0.146
Help to cope with not smoking during school hours and smoking cessation assistance (n=373)	1.10 [0.80-1.50]<br>0.531	1.03 [0.85-1.27]<br>0.698	1.46 [0.96-2.25]<br>0.075	1.06 [0.84-1.30]<br>0.568	0.04 [-0.01-0.12]<br>0.109
Student level - time 2					
New school-break facilities (n=1440-1448) *	1.01 [0.86-1.00]<br>0.379	**1.22 [1.10-1.36]<br>0.000**	**1.20 [1.01-1.44]<br>0.039**	1.24 [1.11-1.39]<br>0.000	**0.07 [0.03-0.11]<br>0.000**
Smoke-free signage (n=1440-1448) *	**1.39 [1.22-1.58]<br>0.000**	0.92 [0.85-1.00]<br>0.078	1.15 [0.98-1.35]<br>0.068	0.93 [0.85-1.02]<br>0.150	0.02 [-0.01-0.05]<br>0.31
Help to cope with not smoking during school hours and smoking cessation assistance (n=393-396) *	0.97 [0.70-1.36]<br>0.9	0.89 [0.72-1.10]<br>0.315	1.16 [0.72-1.89]<br>0.523	1.09 [0.88-1.36]<br>0.402	-0.02 [-0.10-0.05]<br>0.56

** Variations in N is due to missing values in independent variables. Also, at T1 (n=102) respondents from school 1 were missing on the 'quality of delivery' outcome variable.*

Table S7: Unadjusted associations between intervention activities and implementation fidelity of the smoke-free school hours policy, across T1 and T2, at staff/manager level.

	Odds Ratio (OR) with 95% confidence interval (95% CI) P-value				Linear effect with 95 CI P-value
**Intervention activities**	**Adherence**	**Dose**	**Quality of delivery**	**Participant responsiveness**	**Total implementation fidelity***
Staff/manager level - time 1					
Joint workshop before policy implementation (n=184)	1.25 [0.55-2.83] 0.589	1.05 [0.72-1.51] 0.786	1.14 [0.80-1.62] 0.45	**1.75 [1.20-2.54] 0.003**	**0.16 [0.04-0.28] 0.006**
Internalization of fixed enforcement procedures (n=411-419) *	**1.83 [1.23-2.73] 0.002**	1.11 [0.91-1.35] 0.287	1.15 [0.94-1.41] 0.170	**1.60 [1.31-1.97] 0.001**	**0.19 [0.12-0.26] 0.000**
Experienced support from NGOs and local municipality (n=411-419) *	1.15 [0.74-1.74] 0.511	1.11 [0.88-1.40] 0.374	1.16 [0.92-1.47] 0.189	**1.50 [1.18-1.90] 0.001**	**0.14 [0.06-0.22] 0.000**
Staff/manager level - time 2					
Joint workshop before policy implementation (n=134)	1.18 [0.40-3.45] 0.756	1.15 [1.76-1.79] 0.514	0.98 [0.65-1.50] 0.957	**1.72 [1.09-2.71] 0.018**	**0.14 [0.007-0.28] 0.041**
Internalization of fixed enforcement procedures (n=436-452) *	**1.55 [1.06-2.25] 0.021**	1.20 [0.97-1.49] 0.083	1.04 [0.85-1.28] 0.662	**1.64 [1.32-2.05] 0.000**	**0.17 [0.10-0.23] 0.000**
Experienced support from NGOs and local municipality (n=436-452) *	0.79 [0.48-1.31] 0.324	1.01 [0.77-1.33] 0.904	1.12 [0.87-1.45] 0.365	1.09 [0.83-1.43] 0.511	0.14 [-0.05-0.12] 0.422

**Variations in N is due to missing values in independent variables.*
