# Supplementary material for: Intervention Activities Associated with the Implementation of a Comprehensive School Tobacco Policy at Danish Vocational Schools: A Repeated Cross-Sectional Study
Source: Int J Environ Res Public Health. 2022 Sep 30;19(19):12489. doi: 10.3390/ijerph191912489 (PMC9565121; doi:10.3390/ijerph191912489)
Supplement: Supplementary file 1 [file ijerph-19-12489-s001.zip › Table S8.pdf]

8. Sensitivity analysis – associations between intervention activities and implementation fidelity stratified by smoking status

Table S8: Student level associations between intervention activities and implementation fidelity of the smoke-free school hours policy, stratified by smoking status **at T1.**

	Odds Ratio (OR) with 95% confidence interval (95% CI)		*p*-value interaction
	**Smokers**	**Nonsmokers**	
**Adherence**			
Smoke-free signage (N=1189)	**2.12 [1.34-3.35]**	**0.47 [0.29-0.74]**	**0.001**
New school-break facilities (N=1189)	1.20 [0.80-1.78]	0.83 [0.80-1.78]	0.663
**Dose**			
Smoke-free signage (N=1189)	0.98 [0.80-1.20]	1.01 [0.82-1.24]	0.886
New school-break facilities (N=1189)	0.88 [0.68-1.14]	1.13 [0.87-1.46]	0.339
**Quality of delivery**			
Smoke-free signage (N=1086)	1.00 [0.68-1.48]	0.99 [0.67-1.46]	0.978
New school-break facilities (N=1086)	0.92 [0.58-1.48]	1.07 [0.67-1.71]	0.758
**Participant responsiveness**			
Smoke-free signage (N=1189)	1.12 [0.90-1.40]	0.88 [0.70-1.10]	0.286
New school-break facilities (N=1189)	1.16 [0.89-1.51]	0.85 [0.65-1.12]	0.261
**Total implementation fidelity**	Linear regression: B with 95%CI		
Smoke-free signage (N=1086)	0.04 [-0.02-0.12]	-0.04 [-0.11-0.02]	0.230
New school-break facilities (N=1086)	-0.01 [-0.09-0.07]	0.01 [-0.07-0.09]	0.819

NB: The activity 'Help to cope with not smoking during school hours and smoking cessation assistance' was not included in this analysis, as non-smokers were unable to report on this intervention activity.

Table S8: Student level associations between intervention activities and implementation fidelity of the smoke-free school hours policy, stratified by smoking status **at T2.**

	Odds Ratio (OR) with 95% confidence interval (95% CI)		*p*-value interaction
	**Smokers**	**Nonsmokers**	
**Adherence**			
Smoke-free signage (N=1406)	0.82 [0.62-1.09]	1.20 [0.90-1.59]	0.194
New school-break facilities (N=1406)	0.88 [0.62-1.23]	1.13 [0.62-1.23]	0.471
**Dose**			
Smoke-free signage (N=1401)	1.02 [0.84-1.23]	0.97 [0.80-1.28]	0.809
New school-break facilities (N=1401)	1.00 [0.78-1.27]	0.99 [0.78-1.26]	0.978
**Quality of delivery**			
Smoke-free signage (N=1406)	1.01 [0.69-1.46]	0.98 [0.68-1.43]	0.951
New school-break facilities (N=1406)	1.03 [0.66-1.61]	0.96 [0.61-1.50]	0.880
**Participant responsiveness**			
Smoke-free signage (N=1406)	1.03 [0.84-1.25]	0.96 [0.79-1.17]	0.754
New school-break facilities (N=1406)	1.12 [0.86-1.44]	0.89 [0.69-1.15]	0.381
**Total implementation fidelity**	Linear regression: B with 95%CI		
Smoke-free signage (N=1406)	-0.01 [-0.08-0.06]	0.01 [-0.06-0.08]	0.753
New school-break facilities (N=1406)	0.001 [-0.08-0.09]	-0.003 [-0.09-0.08]	0.972

NB: The activity 'Help to cope with not smoking during school hours and smoking cessation assistance' was not included in this analysis, as non-smokers were unable to report on this intervention activity.
